# Advance Directive Documentation in a Huntington’s Disease Clinic: A Retrospective Chart Review

**DOI:** 10.5334/tohm.676

**Published:** 2022-02-01

**Authors:** Christa S. Cooper, Deborah A. Hall

**Affiliations:** 1 Rush University Medical Center, Chicago, IL, US

**Keywords:** Huntington’s disease, advance care planning, power of attorney, advance directives, movement disorders, electronic medical records

## Abstract

**Background:**

Advance care planning (ACP) benefits patients and caregivers, yet it is underutilized and little is known about ACP in Huntington’s disease (HD) clinics. This study sought to determine the percentage of charts with AD documentation within an HD clinic.

**Methods:**

A retrospective chart review was conducted on a randomly selected sample of charts within an HD clinic. HD patients ≥18 y/o with a positive genetic test (≥40 CAG repeats) seen between January 2018 and June 2021 were included. Charts were reviewed for documentation of ADs either in provider notes or in the electronic medical records (EMR).

**Results:**

Ninety-one charts were reviewed (n = 91). Twenty-two charts (24.2%) mentioned a completed AD within a provider’s note; however, only nine (9.9%) had an AD available in the EMR. Cognitive status, primary insurance type, presence of dysphagia, and stage of disease were associated with documentation of completed ADs within a provider’s note.

**Discussion:**

The rate of completed ADs mentioned in a provider’s note (24.2%) was significantly lower than rates of AD completion in a previous study within the HD population (38%). Additional studies focused on improving rates AD completion are needed.

**Highlights:**

Most patients with Huntington’s disease do not have documentation of completed advance directives (ADs) within their medical chart. In a retrospective chart review 24.2% of patients seen in a specialty HD clinic had documentation of ADs in a provider’s note and 9.9% had ADs available within the EMR.

## Introduction

Advance care planning (ACP) is a process that allows patients the opportunity to share their life values, preferences for medical treatment, and wishes for end-of-life (EOL) care. It is universally recommended for individuals with life-limiting diseases and needs to be completed before loss of mental capacity occurs. ACP has numerous benefits including improving EOL experiences for patients and families, improving communication between patients, families, and healthcare professionals, and better concordance between the patient’s preferred medical treatment and medical care delivered [1]. ACP is a patient-centered process that allows patients to not only express their wishes for future medical care but also to appoint a surrogate decision-maker. Healthcare Power of Attorney (POA), Practitioner Orders for Life Sustaining Treatment (POLST), living wills, and do-not resuscitate orders are examples of documents that can be completed during the ACP process and are collectively referred to as advance directives (ADs) [2]. In addition to benefits to individual patients, completion of ADs can also benefit healthcare systems. Patients that engage in ACP and sign ADs save an estimated $9,500 in medical costs compared to those that do not [2]. Savings are due to differences in medical treatment received near the EOL with ACP leading to decreased use of high-cost medical care such as inpatient admissions [2]. Even though numerous benefits of ACP have been identified the rates of individuals engaging in ACP are low. Approximately 36% of the general public completes any type of AD with similar rates of completion between those with chronic illnesses (38%) and healthy individuals (33%) [3]. Older age and poor health have been found to increase the likelihood of engagement in ACP discussions [4].

Huntington’s disease (HD) is a progressive, neurodegenerative disorder that can impact a person’s physical, psychiatric, and cognitive functioning. It is passed down through an autosomal dominant pattern with children of an affected parent having a 50% risk of inheritance [5]. Pre-symptomatic and confirmatory genetic testing has been available for over 25 years but only 15% of individuals at risk decide to undergo genetic testing before the manifestation of symptoms [5,6]. The typical age of onset of symptomatic HD is between 35–45 years old with progression over the course of 15–20 years, ultimately leading to disability and death [5]. Given the fatal and relentlessly progressive nature of HD all individuals affected should engage in ACP and complete ADs. However, in clinical practice only 31–38% of HD patients report completing ADs even though 75% of HD patients express having some thoughts or wishes for their EOL care [7,8].

Currently, there is no standardized model for ACP in the HD population. The literature supports “early” ACP discussions, particularly before the onset of dementia, but the exact timing is not agreed upon [9]. Initiating ACP immediately after receiving a positive pre-symptomatic test result would be challenging given the uncertainly of when symptoms would manifest and discussions about future disability may be difficult to navigate in a currently healthy individual [10].

The purpose of this study was to examine the nature of AD documentation and percentage of patients with documented ADs within a specialty HD clinic. At the time of the study there was no standardized ACP protocol. “Usual care” consisted of broaching the topic of ACP during a routine clinic visit and providing ACP information packets and ADs to patients and caregivers. Documentation of ADs was defined in two ways, either mention of a completed AD, such as POA or POLST, in an HD provider’s note or any completed ADs available in the electronic medical record (EMR). Additionally, we examined if there was a relationship between demographic predictors, co-morbidities, and the completion of ADs. As our institution is planning to implement an ACP intervention, our objective was to determine baseline rates.

## Methods

This was an institutional review board-approved retrospective chart review. The HD clinic is part of an academic medical center in a large urban area. The clinic serves approximately 150 HD patients and their families. There are two movement disorder neurologists, two movement disorder fellows, one physician assistant, two neuropsychologists, one psychiatrist, one genetic counselor, and one social worker involved in the care of HD patients. Included were patients seen in the HD clinic between January 1, 2018 and June 15, 2021, who were at least 18 years old, and had positive genetic testing for HD (CAG repeat ≥ 40). Charts were selected using simple random sampling techniques using the random number method. Demographics collected included sex, age, race, ethnicity, primary language, marital status, years of education, number of years since positive genetic test result, level of cognitive impairment, insurance status and type, presence of dysphagia, and stage of disease. Stage of disease was determined using the Huntington’s Disease Society of America definitions including pre-symptomatic (stage 0), early (stage 1), middle (stage 2), and late (stage 3). A genetic test is considered positive for HD when at least one CAG repeat is ≥ 40, intermediate and indeterminate CAG repeat lengths (27–39) were excluded. Those charts that did not have a specified number of years of education were assigned 13 years for “some college”, 16 years for bachelor’s degree, 18 years for master’s, and 22 years for doctoral degrees. Cognitive status was determined by reviewing neuropsychological testing results in each chart. HD patients in this clinic routinely have baseline cognitive testing and repeated testing when there are perceived changes in cognitive abilities. Patient charts that indicated self-reported cognitive impairment, but no neuropsychological testing, were excluded from this study.

Patient charts from January 1, 2018 to June 15, 2021 were reviewed for evidence of AD completion. We defined ADs as “complete” if 1) completion of any type of AD was mentioned in an HD provider’s note or 2) a completed AD could be found in the EMR. If an AD was available, we noted the type of AD completed (POA, living will, POLST, etc). Data from the chart review was documented within an excel spreadsheet. For analysis, SPSS was used to calculate descriptive statistics on all variables. To assess potential predictors of AD completion data was separated into two categories 1) those who had completed ADs (either mentioned in provider’s note or in the EMR) or 2) those who had no evidence of completed ADs. Sample size estimate was based on a hypothesized 38% completion of ADs with 5% margin of error and 95% confidence interval. Two-sample t-tests were used for analysis of continuous variables including age, number of years since genetic testing, and years of education. For categorical variables chi-square or Fischer’s exact tests were used.

## Results

Ninety-one charts meeting the inclusion criteria were randomly selected from the EPIC reporting database and reviewed. The average patient age was 51.23 years old. The majority of patients were women (56%), white (87.9%), and non-Hispanic (92.3%). This is comparable to our site’s data for the observational study, Enroll-HD, where participants with HD were most commonly women (52.4%), white (89.1%), non-Hispanic (95.1%) and the average age was 50.8 years old. The primary language for most patients was English (93.4%) and over half were married (53.9%). Most patients had some form of cognitive impairment such as mild neurocognitive impairment (33%) or dementia (48.4%). Eighty-six patients (94.5%) had insurance coverage, with Medicare (40.7%) and commercial insurance (40.7%) being the two most common types. Most patients did not have dysphagia (61.5%) and were in the moderate stage (stage 2) of HD (55%). The average number of years since undergoing genetic testing for HD was 7.96 years and the average years of education was 14.15. Sample patient demographics are summarized in ***Table 1***.

**Table 1:** Sample Patient Demographics.


DEMOGRAPHIC	PATIENTS, N = 91

Age, mean (SD)	51.23 (13.81)

Years of education, mean (SD)	14.15 (2.44)

Sex, n (%)	

Men	40 (44%)

Women	51 (56%)

Race, n (%)	

White	80 (87.9%)

Black	5 (5.5%)

Asian	1 (1.1%)

Other	5 (5.5%)

Ethnicity, n (%)	

Non-Hispanic	84 (92.3%)

Hispanic	7 (7.7%)

Primary Language, n (%)	

English	85 (93.4%)

Spanish	4 (4.4%)

Korean	1 (1.1%)

Polish	1 (1.1%)

Marital Status, n (%)	

Single	27 (29.7%)

Married	49 (53.9%)

Divorced	14 (15.6%)

Widowed	1 (1.1%)

Stage of Disease, n (%)	

Pre-symptomatic	13 (14.3%)

Early	15 (16.5%)

Moderate	50 (55%)

Late	13 (14.3%)


**Figure 1:**
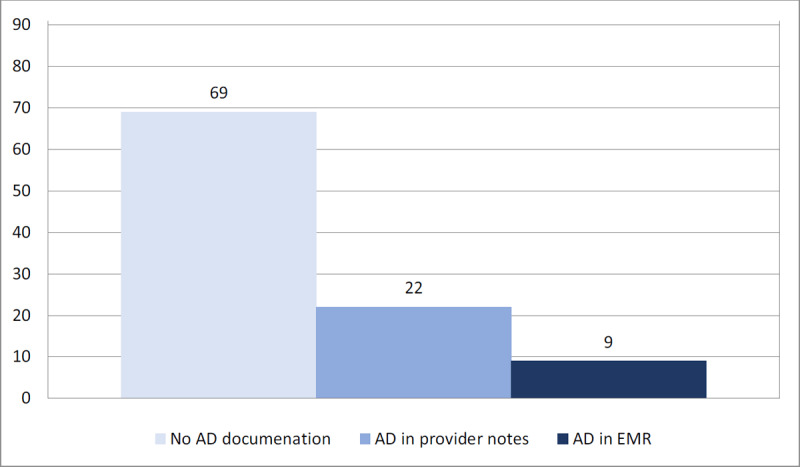
AD Documentation Type.

***Figure 1*** displays the variation in AD documentation among the HD clinic sample. There is some overlap between patients that had documentation in a provider’s note and who had ADs in the EMR. Twenty-two of 91 charts reviewed (24.2%) had documentation of ADs within a provider’s note, but only nine of 91 (9.9%) had ADs scanned into the EMR. The most common type of AD mentioned in a provider’s note and available in the EMR was POA. All 22 charts (100%) mentioned completion of POA in a provider’s note and all 9 (100%) had POA available in the EMR. One patient’s chart had a living will document available in the EMR and eight provider’s notes mentioned other types of ADs. Most ADs were scanned into the “media” section of the EMR system as opposed to other areas of the EMR. Seventy-five percent of the charts reviewed had no evidence of completed ADs.

Post-hoc tests revealed significant differences between patients that did not have ADs and those that had ADs documented in a provider’s note. These differences were significant in cognitive status (p = 0.02), primary insurance type (p = 0.01), presence of dysphagia (p = 0.02), and stage of disease (p = 0.003). Patients with dementia, dysphagia, Medicare, and moderate or late-stage disease were more likely to have ADs documented in a provider’s note. Relationship between age, race, ethnicity, marital status, numbers of years since genetic testing, years of education and completion of ADs were not significant (p > 0.05). Patient characteristics related to AD documentation are summarized in ***Table 2***.

**Table 2:** Patient Characteristics of AD Documentation.


PATIENT CHARACTERISTIC	NO ADVANCE DIRECTIVE DOCUMENTATION (N = 69)	ADVANCE DIRECTIVE DOCUMENTED IN PROVIDER NOTE (N = 22)	P-VALUE

# of years since HD genetic testing, mean (SD)	7.38 (6.15)	9.77 (6.08)	0.11

Presence of dysphagia n, (%)			

No	47 (68.1)	9 (40.9)	0.02*

Yes	22 (31.9)	13 (59.1)*	

Cognitive Impairment, n (%)			

None	16 (23.2)	1 (4.6)	0.02*

MCI	25 (36.2)	5 (22.7)	

Dementia	28 (40.6)	16 (72.7)*	

Insurance status, n (%)			

Not insured	4 (5.9)	1 (4.6)	1.0

Insured	65 (94.2)	21 (95.5)	

Primary insurance type, n (%)			

Commercial	34 (49.3)	3 (13.6)	0.01*

Medicare	21 (30.4)	16 (72.7)*	

Medicaid	9 (13)	2 (9.1)	

VA	1 (1.5)	0	

N/A	4 (5.8)	1 (4.6)	

Stage of Disease, n (%)			

Pre-Symptomatic	13 (18.8)	0	0.003*

Early	14 (20.3)	1 (4.6)	

Moderate	36 (52.2)	14 (63.6)*	

Late	6 (8.7)	7 (31.8)*	


MCI = Mild Neurocognitive Impairment, VA = Veterans Affairs insurance. * Denotes significance.

## Discussion

Our study found 69 of 91 charts (75.8%) reviewed did not have any evidence of ADs either in a provider’s note or within the EMR. Another study analyzing AD completion in the HD population involved online patient and caregiver surveys. The authors found a completion rate of 38.2% which is significantly higher than our rate of 24.2% (p = 0.01) [8]. Our results may be lower than actual rates of AD completion in the HD population if patients have completed ADs but have not shared it with their HD provider or if the HD provider did not document completion of ADs in their notes. In contrast, the online survey study did not define ADs, living wills, or other ACP terminology so it is possible patients and caregivers answered without fully understanding their meanings causing an artificially inflated result. Furthermore, the online survey may have been prone to selection bias, was not validated, and relied on self-reported completion of ADs so this data may not be a true indication of prevalence. Another retrospective chart review for AD completion in a geriatric primary care clinic found 25.5% of geriatric patients had documentation of ADs in a provider’s note which is similar to our study (p = 0.86) and did not rely on self-reported data [11].

There was no standard protocol for broaching the topic of ACP within the HD clinic at the time this study was conducted therefore it is possible that patients and caregivers had completed ADs and providers were just unaware. Another possibility is that HD patients had filed ADs with another provider, such as their internist or family medicine physician, at a different site. Although there is some sharing between EMR systems not all hospitals or private practice medical offices EMRs are connected, therefore, it is imperative that HD patients provide copies of ADs to all of their healthcare providers. Furthermore, given the complexities of late-stage HD it is most appropriate for medical professionals experienced with treating HD patients near the EOL to engage in ACP with this population. This study will be used as a baseline before the adaptation of a new workflow to address ACP with our HD patients.

Our study found that patients with moderate and late-stage HD were more likely to have documented ADs in a provider’s note. There may be several factors influencing these results. First, patients in moderate and late-stage disease are closer to EOL and have had more time to think about their EOL wishes compared to those in pre-symptomatic or early stages. Given the progressive nature of HD, patients in later stages are more likely to have higher symptom burden, such as dementia and dysphagia which were also correlated to increased AD documentation in our study. Onset of dysphagia and dementia may trigger patients, their caregivers, or HD providers to broach the topic of ACP. Secondly, HD providers may be more likely to ask about ADs in patients that have progressed to moderate and late-stage HD and therefore documentation in the EMR would increase. Future studies could evaluate what prompts an HD provider to discuss ACP and ADs with their patients.

Participants in our study with Medicare were more likely to have completed ADs documented in a provider’s note. Medicare coverage typically starts when an individual turns 65, however, many HD patients are eligible for Medicare at a younger age if they have been on disability for at least 2 years. This means that HD patients on Medicare are likely older or more disabled than patients covered by other insurance plans. Older age has been associated with having completed ADs in previous studies [8].

There is often confusion regarding who should initiate ACP discussions as well as when these conversations should be started. A recent systematic review identified barriers to ACP at the individual, interpersonal, provider, and system level [12]. Examples of barriers included lack of knowledge of ADs, concern for jeopardizing the patient-provider relationship, misunderstanding of who should initiate the discussion, poor family relationships, time pressures, and lack of clarity on the ACP process itself [12]. Although the providers in the HD clinic are qualified to discuss ACP with their patients, lack of time and uncertainly on when to start ACP discussions were cited as major barriers.

All patients with a completed AD mentioned in a provider’s note or scanned AD available in the EMR had POA. POA forms are the most common AD document given to patients and caregivers in this HD clinic which likely led to these results. Identifying a surrogate decision-maker is an important step in the ACP process but is not the only component that should be addressed. It was not clear in provider’s notes or in the EMR if surrogate decision-makers were aware of patients’ EOL preferences or wishes for medical care. Completing POA paperwork does not guarantee that patients and caregivers have discussed how to handle future medical scenarios or decisions such as feeding tubes, CPR, or other life-sustaining treatments. This is a major missing component of ACP as these discussions are important for both patients and surrogate decision-makers. It is possible that patients and caregivers had these discussions when completing POA paperwork and it was just not documented. Some states include probing questions about future medical wishes and life-sustaining treatments in their POA forms but in Illinois (where this clinic is located) those questions are optional.

Finding ADs within the EMR system was also somewhat complicated as there were multiple different areas within the EMR where ADs could be stored. Some charts actually had ADs available in the EMR, however, under “code status” in the EMR it incorrectly stated, “no ACP docs.” Having multiple locations within an EMR system to search for ADs is cumbersome and may result in inaccessibility of ADs when clinically needed. Provider notes were also not consistent with where ADs were mentioned and there were some conflicting notes. One patient stated they had completed ADs with their family member, and this was documented in the provider’s note. At the next visit the patient reported they had actually not completed any ADs or ACP discussions, but they were planning to do so. The provider updated their note to reflect this new information. Since provider notes reflect what the patient or caregiver report it is possible that the information being reported is inaccurate. The inconsistency in provider notes made it impossible to determine the quality of ACP discussions that may have occurred or if they occurred at all. Our clinic is using this information to improve the rates of HD patients and caregivers engaging in ACP discussions, the quality of these discussions, and completion rates of ADs. Establishing a workflow for consistent documentation of ACP discussions and completed ADs within provider notes has the potential to improve the ACP process. Engaging more HD patients and caregivers in the ACP process will allow them to reap the many benefits of ACP. Lack of time was identified as a major barrier to provider’s ability to broach ACP during clinical visits; therefore, options to have dedicated visits where the only objective is to discuss ACP is a topic of interest.

The purpose of this study was to determine baseline rates of AD completion and documentation within an HD clinic and to assess demographic predictors of AD completion. This study will be utilized to help establish quality improvement projects regarding ACP within an HD clinic. It will also help promote future research on ACP within other movement disorder clinics which may be facing similar barriers such as lack of time to address ACP and disorganized documentation of ADs within provider notes and EMR systems. Next steps include the implementation of a new workflow for ACP and to conduct follow up studies after implementation to assess impact on completion and documentation of ADs. Similar methods for retrospective chart review can also be utilized to determine baseline rates of AD completion in other movement disorder clinics.
